# Immediate Root Filling

**Published:** 1888-01

**Authors:** 


### IMMEDIATE ROOT FILLING.

There is a curious lack of comprehension of the principles involved in a practice now urged by many dentists—that of immediate root filling. When Dr. Harlan, at the last meeting of the American Dental Association, condemned this method of procedure, and at the same time stated that he probably filled sixty per cent. of his cases without prolonged treatment, he was upbraided with inconsistency. A usually very intelligent contemporary says that between those who condemn the practice of immediate root filling, and yet admit that in a majority of cases they practice it, and those who advocate the doctrine, there exists "a distinction without a difference." This is certainly an error. There is no difference of opinion concerning the propriety of filling aseptic canals immediately, and these probably comprise more than half the cases in practice. Nor is there any very serious questioning of the practice of the immediate filling of canals where fistula exists. It is concerning the method of procedure with septic roots—of so-called blind abscesses—that a difference of opinion is expressed. No one questions the advisability of, usually at least, immediately filling a canal that is in a healthy state, save for the mere traumatism of the removal of a pulp. All that prolonged treatment can do is to reduce to a normal condition, as far as inflammation and septicism are concerned, and if neither has ever existed in the tissues surrounding the tooth, surely the first state is as favorable for filling as it can be subsequently. There may be a difference of opinion about the condition of the living portion of the dentine, but so far it has not prominently entered into the discussion of the subject.

The advocates of immediate root filling urge it as applicable in all cases—in canals that have long been in a septic state without other drainage than that through the cavity of decay, in cases of active putrefaction of the pulp, as well as in teeth from which the pulp has but just been removed—and it is here where issue is joined. Those who practice immediate filling are strong in the faith of the bacterian origin of all septic conditions. Their method of procedure in a case of blind abscess is practically this: The rubber dam is so adjusted as entirely to exclude saliva, and access to the pulp canal is obtained. A disinfectant—preferably peroxide of hydrogen—is then introduced into the canal, either by means of a delicate syringe or by a few fibers of cotton wound on a broach, and this is forced into the canal until all signs of septic products have ceased. This is usually determined, if peroxide of hydrogen is used, by the cessation of effervescence; if other agents, by the fact that they do not change in color or appearance. An instrument is then used to open the canal still further. A Morey drill is introduced, or the canal is enlarged, cleared and opened yet further by the excellent Donaldson canal cleaners, when the disinfectant is again employed, and this is continued, if possible, until the apex is reached and the agent is carried through the foramen. When entirely satisfied that the territory is disinfected, a germicide, like mercuric chloride, is introduced and carried to every portion, and the canal is dried and filled at once.

There is much that commends itself to the judicious operator in this method. The reasoning of its advocates is, that as the diseased condition is entirely due to microbes, when they with their products are destroyed and removed there is no excuse for prolonging treatment, and the process of repair should be at once commenced, and this will be best secured by the absence of all medicaments and the permanent closing of the usual source of infection. In most cases this is probably true, but we think there are one or two points that are overlooked. In many cases of blind abscess of long standing there is an infiltration of septic matter into the territory surrounding the original point of infection. Not infrequently there is osteitis of a more or less pronounced character, with a deposition of inflammatory products that have assumed, perhaps, an indurated character. There is a low chronic form of this inflammation extending for some distance into the surrounding tissues, and something beside a mere disinfectant or germicide is required to promote the absorption of the depositions. In cases of induration, as in all abnormal growths, there are but two methods by which nature can remove them. The one is by absorption, and the other by a breaking down of the tissues and sloughing. We desire to bring about the former and to guard against the latter. The readiest and most convenient, as well as the most effectual method of reaching the disturbed territory, is through the root canal, and if this be closed we must rely, in case the inflammatory products are not at once removed, upon medication by absorption through the gum, or upon surgical interference. The latter is to be deprecated unless all other means fail. The gums consist of fibro-cartilaginous tissue and do not readily absorb medicaments. So that if there be, as too often there is, a deposition of indurated lymph, the pulp canal forms the natural passage to the centre of the point of disturbance, and should be kept open for the introduction of the proper stimulants.

Again, when the septic organisms have worked their way back into the tissues in case of a blind abscess, it is extremely difficult, if not impossible, to reach the farthest point of infection at a single sitting. We must often trust to absorption and slow infiltration of the antiseptic to reach the extremest point. The tooth tissue, too, may contain septic fields which can only be reached by gradual penetration. There is no security that a single application will make all the tissues entirely aseptic. The process of repair should be commenced, at least, before the whole is permanently sealed up. If an acute pericementitis succeeds the cleaning of the root-canal, it is much more easily treated if the canal be open than if it be closed. Hence, we can readily see that there exists a certain class of cases in which common prudence would demand that we proceed with due caution. The proper treatment of these requires, upon the part of the operator, not only caution, but a thorough knowledge of the pathology involved. Every operator should, of course, at once proceed to disinfect them if they are septic, and to introduce a proper germicide. But once made aseptic, the method of procedure should be changed. Violent drastic antiseptics should no longer be employed, but the whole attention should be directed toward the removal of the inflammatory products, and the healing of the cavity left in the tissues, antiseptics proper being employed only to keep the whole in an aseptic state. Not infrequently have we filled the root-canal with pure tincture of iodine to stimulate absorption, and under its influence seen an induration that exhibited itself upon the outside of the jaw rapidly melt away. As a resolvent, we know of nothing that is more prompt in its action.

Much has been said concerning the use of coagulators of albumen in the treatment of dead teeth, and we believe that undue stress has been laid upon what is practically of little moment. The most of the remedies commonly employed are coagulators, and we fail to see any objection, on this account, to their proper use. Albumen is coagulated spontaneously upon the admission of air, and any which may exist in an unorganized or inactive state will coagulate in any case. We would not introduce a coagulant into pus, unless at the same time it was broken up and provision made for its removal. In a tooth that is in a septic state there can be no protoplasmic albumen in the dentinal tubules, the mouths of which might, by coagulation, be obstructed and the entrance of an antiseptic influence thus prevented, and hence we fail to comprehend what practical danger there may be in the use of coagulating remedies. An antiseptic, whether a coagulant or not, should be introduced only when disinfection and the removal of septic products is as complete as possible, and our choice of a remedy then, whether it be carbolic acid, iodoform, permanganate of potassium or mercuric chloride, would depend upon something more than its power to coagulate albumen.

In cases in which a fistulous opening through the gum exists, immediate root-filling may often, perhaps always, be permissible. There is perfect drainage through the fistula, and it is possible to reach the point of infection, if necessary, through it. There is a question, however, whether it be not sometimes better to keep the canal open and to treat through it, especially if there be free communication through the foramen, than to run the risk of a necessity for attempting to follow up the fistula through its usually tortuous course, for sometimes the opening is at a considerable distance from the disease centre. An opening might, of course, be made through the external alveolar plate, but this surgical interference is what we usually desire to avoid.

The summing up of the matter is, then, according to our view, that immediate root-filling is admissible in cases where there is little septic infection or inflammation beyond the apex of the tooth, in most cases of abscess with a fistulous opening, and in the majority of those in which disinfection is positively complete. But when there is probably considerable infiltration or deposition of inflammatory products, or when there is a chronic irritation as manifested by a persistent pericementitis of a sub-acute character, the use of the proper remedies introduced through the pulp-canal should be continued until resolution is either begun or completed.

